# Inguinal hernia surgery in developing countries: should laparoscopic repairs be performed ?

**DOI:** 10.11604/pamj.2017.27.5.12358

**Published:** 2017-05-02

**Authors:** Berthier Nsadi, Olivier Detry, Willy Arung

**Affiliations:** 1 Department of Abdominal Surgery, CUK, University of Kinshasa, Democratic Republic of Congo; 2 Department of Abdominal Surgery and Transplantation, CHU Liege, University of Liege, Liege, Belgium; 3 Department of General Surgery, University of Lubumbashi Clinics, University of Lubumbashi, Lubumbashi, Katanga Province, Democratic Republic of Congo

**Keywords:** Hernia, laparoscopy, mosquito net, mesh, complications, third world, cost

## To the editors of the Pan African Medical Journal

In a recent paper in the Journal, Nana et al. reported their experience with laparoscopic transabdominal pre-peritoneal (TAPP) repair of inguinal hernia [1]. This team from Yaoundé, Cameroon, described the postoperative results of 7 patients operated on between 2011 to 2014. All patients were operated on successfully, and the authors did not report any recurrence or postoperative pain at follow-up. In 2014, in the same Journal, Shakya et al, from Nepal reported their experience of 50 patients operated on for inguinal hernia with both TAPP and total extra peritoneal (TEP) laparoscopic approaches [2]. They also reported good results in terms of recurrence rate and postoperative pain. The authors of these two papers should be congratulated for their efforts to develop laparoscopic procedures in such challenging medical environments. However despite our personal experience of laparoscopic surgery in the Democratic Republic of Congo (DRC) [3, 4], we consider that laparoscopic hernia repair should not be encouraged in developing countries. It has been clearly demonstrated in developed countries that the modern standard of care for inguinal hernia is mesh repair, either through an open repair, namely the Lichtenstein procedure (LP) or through a laparoscopic approach (TEP or TAPP). Compared to non-mesh repairs, the use of a mesh in inguinal hernia surgery provide better results in terms of recurrence and decreased early and late postoperative pain. However the fact that mesh repairs are the modern standard procedures for inguinal hernia poses several issues in developing countries. In these countries, medical meshes are often not even available, as very few commercial companies are importing them there. Secondly, the price of a medical mesh is often unaffordable for the vast majority of patients.Finally, the risk of mesh infection is particularly feared in developing countries [5], even if, in practical, mesh or wound infection after mesh inguinal repair is quite rare. Recently the concept of low-cost mesh has been proposed to overcome these issues: pieces of mosquito nets, a cheap product largely available in developing countries, could be sterilized and surgically used in the same way as commercially available meshes [6].

Randomised clinical studies have shown that this concept might be valid [7]. Another means to decrease the cost of these meshes is also to sterilize polypropylene pieces that are cut from large unused medical meshes. Even if meshes are available, the question of the surgical approach for mesh inguinal repairs is a clear matter of debate in both Western but particularly in developing countries. Compared to LP, TEP and TAPP hernia repairs are challenging surgical procedures with clear learning curves and more possibility of life-threatening complications. Recurrence rates after laparoscopic repair are strongly dependent to the surgeon's experience. Laparoscopic hernia repairs necessarily require general anaesthesia while LP can be quite easily performed under epidural or even local anaesthesia, anaesthetic methods that are well-adapted to developing countries. Laparoscopic surgery necessitates the availability of CO2 and electricity. Laparoscopic surgery requires complicated medical equipment and surgical tools that are quite difficult to sterilize and to correctly maintain in good condition. It is also regularly advocated that laparoscopic repairs cause less postoperative pain than LP, but this matter is still to be debated, particularly in the long term. Finally, it is clear that due to the increased costs of laparoscopic procedures, LP is more cost-effective than laparoscopic repairs, both in Western countries and in the developing world. It should maybe also be considered if LP under local or loco-regional anaesthesia in one-day clinic should not be definitely considered as the standard of care for hernia repair worldwide. In conclusion, from our own experience of laparoscopic surgery in DRC, we strongly believe that there is no reason to develop inguinal laparoscopic repair in developing countries. Laparoscopic repairs are more expensive and more difficult to perform and to learn. The next step of abdominal wall repairs in the developing world should focus on teaching the surgeons to use either commercial or low-cost mosquito meshes in open repairs and assessing the results of these procedures in such challenging medical and surgical environments.

## Competing interests

The authors declare no competing interest.
